# Dental Education

**Published:** 1875-05

**Authors:** Geo. H. Cushing

**Affiliations:** Chicago, Ill.


### DENTAL EDUCATION.

BY GEO. H. CUSHING, D. D, S., CHICAGO, ILL.

*Read before the Mississippi Valley Dental Society, March 4th,* 1875.

It is a very easy thing to criticise methods or systems with the formation of which the critic has had nothing to do, but it is not always so easy to offer remedies for apparent or acknowledged defects which shall prove of practicable application.

Yet thankless and distasteful as the office of critic may be, it must be assumed by any one writing upon the subject of dental education which it is the purpose of this paper briefly to discuss; and a wise and just criticism should tend to advance the interests of the subject under consideration.

This subject has recently engaged and is now engaging the attention of the profession to a greater degree than ever before, since the establishment of dental schools, and a feeling has arisen and is daily growing stronger that the instruction of those seeking to enter the profession is inadequate and unsatisfactory —this feeling finding expression chiefly in censure of the dental colleges.

It will not be attempted, in the brief space allotted to this paper to review the whole field which would have to be traversed in considering fully what, in detail, dental education should be; but reference will be made to some of the evident defects now existing which might easily, and should be, corrected.

The profession at large should correct at once and forever the grave fault of receiving as students persons who have had less than a reasonably good common school educatiou, and who possess less than a reasonable moral character.

They should also refuse to retain as students those who after sufficient evidence prove notably unfitted, by reason of a lack of requisite gifts, for acquiring a respectable position in the profession; and lastly they should receive no students unless they intend to devote such time to their instruction as the stuents have a right to expect, and as their proper qualification at the hands of their preceptors may require. These faults are very grave and very general, and lie at the foundation of this whole matter. It is too generally the case that dentists who are willing to take students at all, make no inquiry either as to their moral character, or their intellectual acquirements, and too often are only actuated in the matter by the consideration of how remunerative they can make their labor, as servants doing the drudgery of the laboratory, while their so called education consists wholly of what they may learn of the mysteries of rubber and cheap dentures. When these practices are amended, there will be better material furnished to the colleges and in consequence so much better men graduated from them.

Now as to the colleges, what are the most apparent faults with them. First, there are far too many of them. If there were in the place of the ten, now in operation in this country, but two, or at the most three, located at the best points for the various sections of the country, and the energies of the profession were concentrated upon the three, we should undoubtedly see better results than are now attained.

It is clearly impossible to obtain the best talent and have it devote enough of its time to secure the proper education of young men for the dental profession, where there are so many schools and so few students. Now are we likely to see any improvement in this direction? It is very much to be feared not, so long as human nature remains human nature! Then accepting as inevitable this most unfortuuate multiplicity of schools, can anything be done to improve the quality of graduates that they send forth? Let us look at some of the other more apparent defects of the system and perhaps it may appear where improvement is possible. The standerd of graduation is too low in our colleges. This is a fact that will hardly be disputed by any, and the system of examination by the professors of the schools is radically wrong. There should be a board of examiners, entirely and absolutely independent of the college and of their faculties, such as the English schools have. Both of these defects could easily, and should, be corrected, and it is hardly possible that the profession will hold the colleges blameless until they see some determined movement to improve these conditions. These changes can easily be made without entailing upon the faculties any greater sacrifices than they now are making, and no excuses or explanations by the authorities of the schools will serve to quiet the demand which is made for, at least the evidence on the part of the colleges of a disposition to advance the standard of dental education. Another great defect in the schools, is the inadequacy of the practical instruction in the operative department. While the principle demand upon the dentist is for operations requiring the greatest dexterity in the use of instruments, and a high degree of manipulative ability which can only come from extended practice, it is very evident that even under the most favorable circumstances, as the colleges are now conducted, graduates must leave their Alma Mater sadly deficient in that practical education which is so essential to qualify them for the duties of their profession. Just how this defect may be remedied is not perhaps so easy to state, but that it should be, is beyond a doubt.

The above are the most obvious defects in our present system of dental education, and some of them, as has been suggested, can easily be remedied.

There is a ground of complaint against the colleges implying a defect in the system some where, which it is difficult properly to characterize. It is intangible except as to its effects, but it is still held to be in some sense a reproach and to call for reform. It is supposed that institutions of learning, like our dental schools, leave their impress upon the character of their graduates. They do leave their impress, for good or evil, so when we see in a single city, within the three years last past, six persons known to be graduates from three of the leading colleges in the land, and two others claiming to be graduates from some of these same colleges—all fresh from their Alma Mater, with whatever impress they have received from their teachings, and associations with faculty and students newly stamped upon them—when we see these young men coming directly from their schools and at once engaging in the most disreputable practices—some of them going at once into the offices of the most notorious quacks as assistants—others starting upon their own account with all the parade and clap-trap of the mountebank—we are inevitably led to conclude that the *morale* of these institutions is not what it should be. This is no fancy picture, nor exaggerated statement, but a plain recital of fact, susceptible of verification at any time. It may be said that this is an exceptional coincidence. It is doubtless an extreme case, yet the same tendency has been observed to a greater or less degree all over the country.

In the same city where the above occurred, but one known graduate, during the same period, established himself in a reputable manner. No explanation will be offered here of why this is so, but it is urged that it clearly is the duty of the faculties of the various colleges to do their utmost to remove such a stain from the reputation of our educational institutions.

There are other improvements that doubtless are very desirable, but these are imperative—easily made, and once established would pave the way for others.

It doubtless may be urged against their practicability, that the schools would not act harmoniously in this matter. It cannot be said that this is so until the trial is made, and we are bound to believe that all the schools have too much at heart the real welfare of the profession, not to readily fall into any general arrangement that shall secure the improvements demanded. If there are any of the schools that would not agree to co-operate in such a movement—let all those who have the right spirit act independently of them and elevate their standard without being frightened or intimidated by the bug-bear of competition, and the time would soon arrive when all would stand upon the same platform.

Doubtless in the discussion of this subject will be heard the oft-told tale of the sacrifices which professors in our dental schools continually make for the welfare of their students. Now probably no one who has not experimentally demonstrated it in his own experience,has a more full appreciation of the extent of these sacrifices than the writer of this paper, and for all the unselfish philanthropy which has been in part the motive power in the lives of such men—they deserve the fullest meed of praise, and no word in this paper is intended to detract in the slightest degree from their well earned glory; but they should remember that they stand in the foremost rank, and are properly looked up to by the profession at large, not only as conservators of its interests, but as pioneers in reform and advancement and they should not pass by unheeded the reasonable demands of the day and the hour.

It has not been the purpose of this paper to fully discuss this subject, but only to open the discussion with such suggestions as seemed of vital importance and of practicable accomplishment. All that has been here said has been, before, much better said, especially in the report on this subject read before the American Dental Association at Nashville. But it is hoped that the reiteration of what has before been urged may occasion that serious consideration which is due the importance of the subject. Doubtless your discussions will bring to bear upon the subject the force of matured thought which shall bring out the many points necessarily comprised in its proper consideration, and it is to be hoped that such an impetus will be given to the foward movement, that we shall soon have cause to rejoice in the fruition of our hopes for a higher dental education.

